# Supplementary figures and images for: Single B cell transcriptomics identifies multiple isotypes of broadly neutralizing antibodies against flaviviruses
Source: PLoS Pathog. 2023 Oct 9;19(10):e1011722. doi: 10.1371/journal.ppat.1011722 (PMC10586629; doi:10.1371/journal.ppat.1011722)

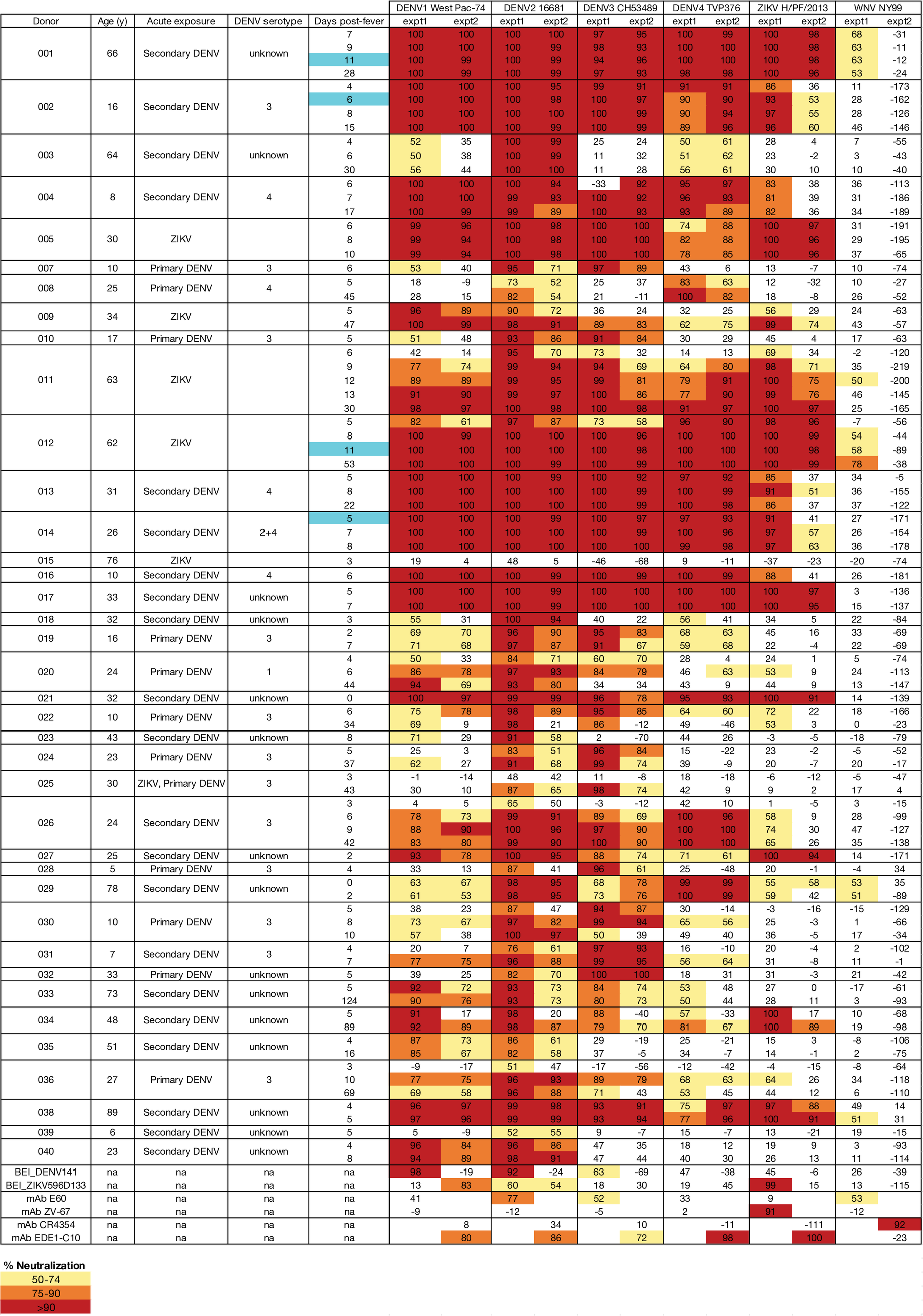

Supplement: S1 Fig — Serum samples from 38 cohort participants with the indicated age and DENV and/or ZIKV acute exposures collected at the time point(s) shown were diluted either 1:240 (expt1) or 1:300 (expt2) and tested for their ability to neutralize the indicated reporter viruses in two independent experiments. Bottom rows indicate control antibodies, which include human convalescent sera to DENV (BEI Resources NR-50232) or ZIKV (BEI Resources NR-50752) and monoclonal antibodies (mAb) E60 [143], ZV-67 [144], CR4354 [54], and EDE1-C10 [28]. The percent neutralizing activity shown under each virus column is normalized to infection in the absence of antibody. Heatmap colors represent neutralizing activity of at least 50% as indicated in the key under the table. We selected corresponding PBMC samples from the donors and time points highlighted in blue under the ‘Days post-fever’ column for single-cell RNA sequencing to isolate monoclonal antibodies. (TIF) [file ppat.1011722.s001.tif]

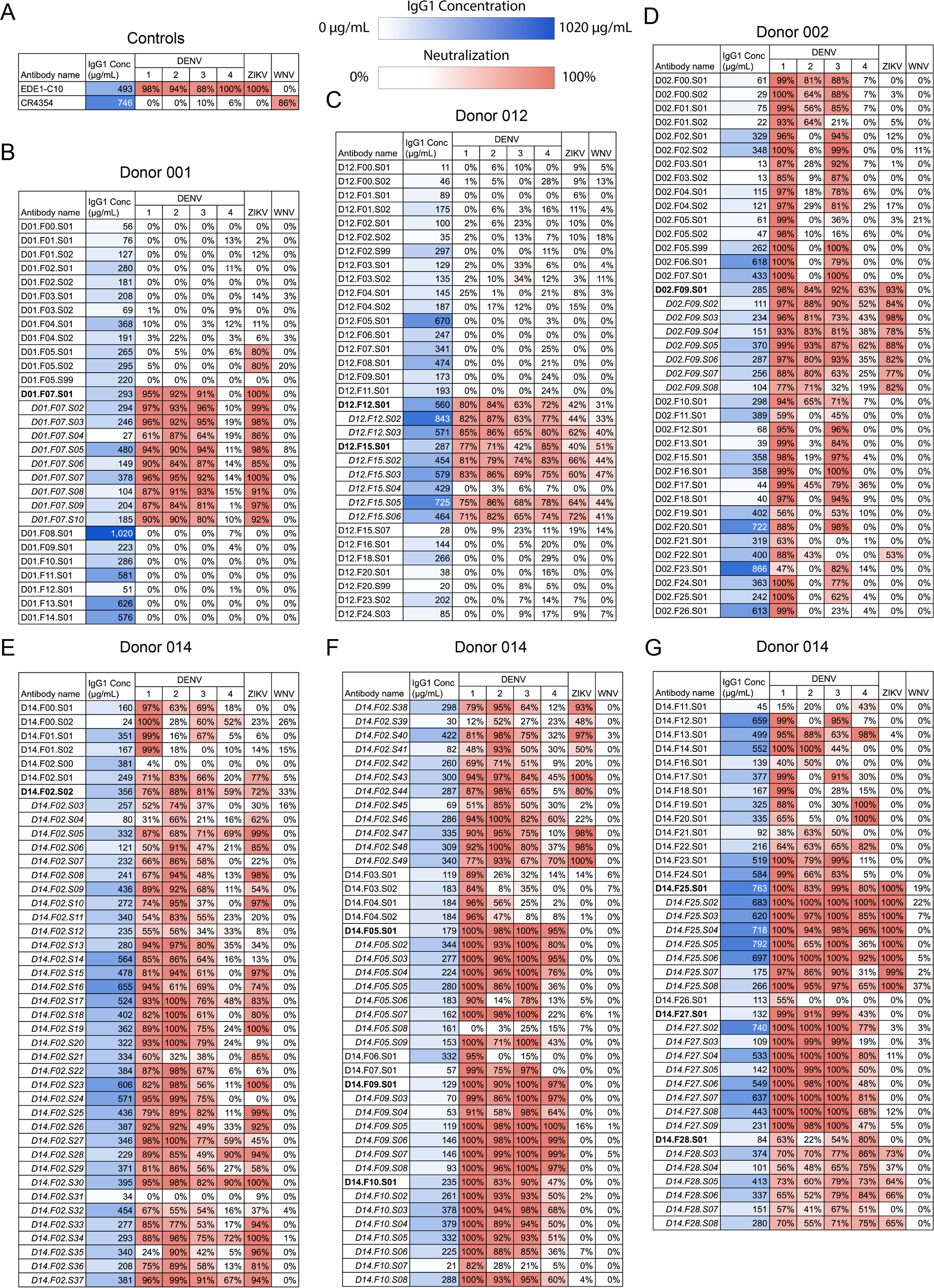

Supplement: S2 Fig — Transfection supernatant containing (A) control antibodies, EDE1-C10 [28,31] and CR4354 [54] or antibodies from donors (B) 001, (C) 012, (D) 002, and (E-G) 014 indicated in each row was tested for neutralization against DENV1 WP-74, DENV2 16681, DENV3 CH54389, DENV4 TVP376, ZIKV H/PF/2013, and WNV NY99 reporter viruses. The second column displays the concentration of IgG1 detected in each crude supernatant as determined by ELISA, displayed as a blue heatmap according to the key. The supernatant composed 1/10 of the volume of each neutralization assay, so the final concentration of antibody present in each assay was 1/10 the value displayed. The red heatmap displays the percent neutralization of each virus normalized to infection in the absence of antibody, as indicated in the key (only values >25% are highlighted in each panel). Antibodies were named based on the source of the antibody in the format DXX.FYY.SZZ, where XX is the donor number, YY is the clonal family within the donor ranked by decreasing size, and ZZ is assigned by the chronological order in which antibodies from the family were produced. Antibodies whose names are left aligned were screened in round 1, which was intended to screen many different families. Antibodies that were considered hits due to the breadth and/or potency of their neutralization in round 1 are shown in bold font. For round 2 we selected additional antibodies, shown indented and italicized, from the clonal families of hits identified in round 1. (TIF) [file ppat.1011722.s002.tif]

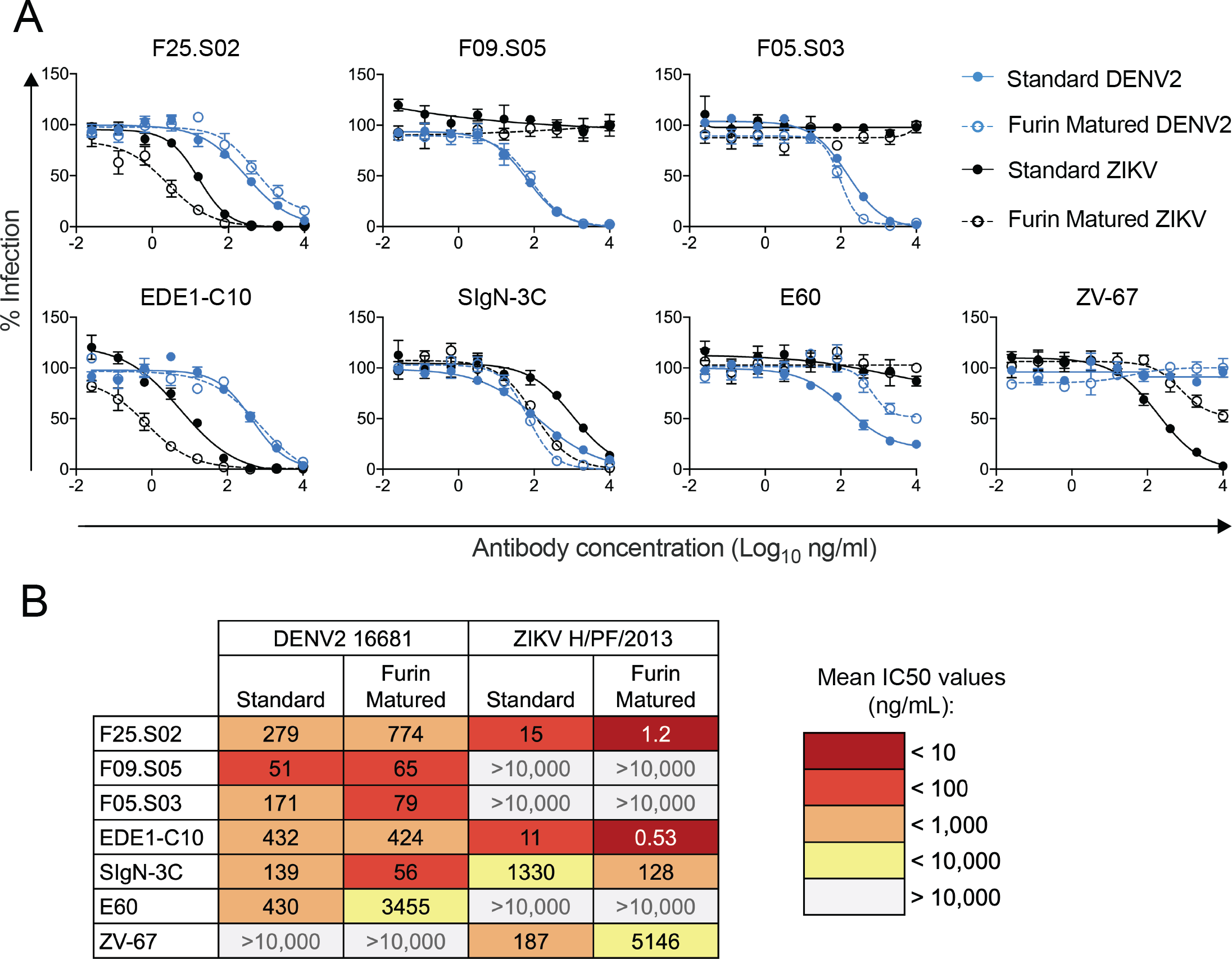

Supplement: S3 Fig — (A) The indicated antibodies were tested against DENV2 16681 (blue) or ZIKV H/PF/2013 (black) reporter virus particles prepared either under standard conditions (solid circles and lines) or in the presence of excess furin (open circles and dashed lines). Data were obtained from two independent experiments, each performed in duplicate wells. Data points and error bars represent the mean infection and standard deviation of the four total replicates, respectively. (B) The table displays the mean IC50 values at which the indicated antibodies neutralized the indicated forms of DENV2 and ZIKV in dose response neutralization curves as shown in (A). (TIF) [file ppat.1011722.s003.tif]

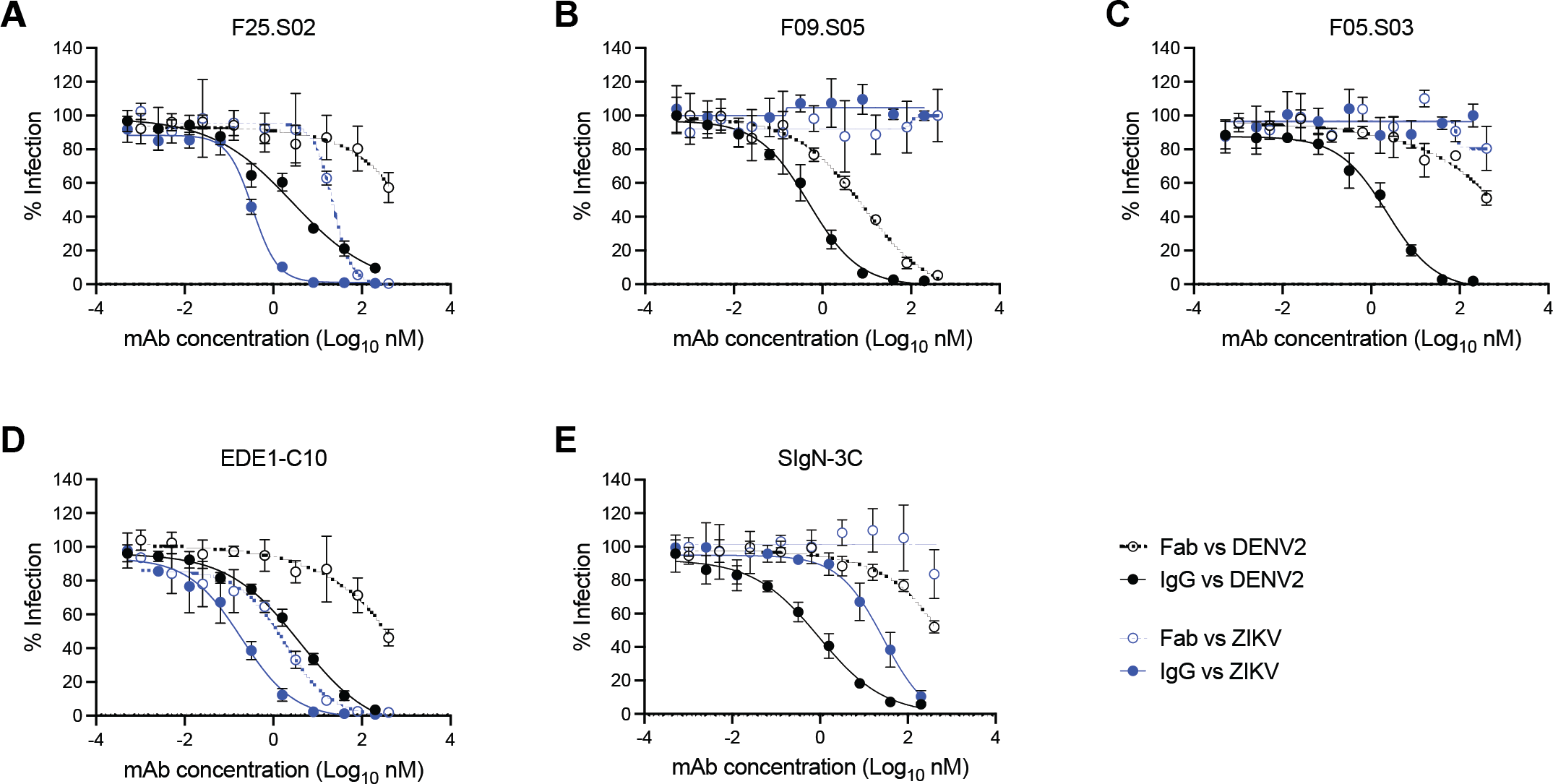

Supplement: S4 Fig — We tested monovalent Fab (open circles and dashed lines) or bivalent IgG1 (solid circles and lines) versions of antibodies (A) F25.S02, (B) F09.S05, (C) F05.S03, (D) EDE1-C10, and (E) SIgN-3C against DENV2 16681 (black) or ZIKV H/PF/2013 (blue) reporter virus particles. Dose-response neutralization curves shown are from two independent experiments, each performed in duplicate wells. Data points and error bars represent the mean infection and standard deviation of the four total replicates, respectively. (TIF) [file ppat.1011722.s004.tif]

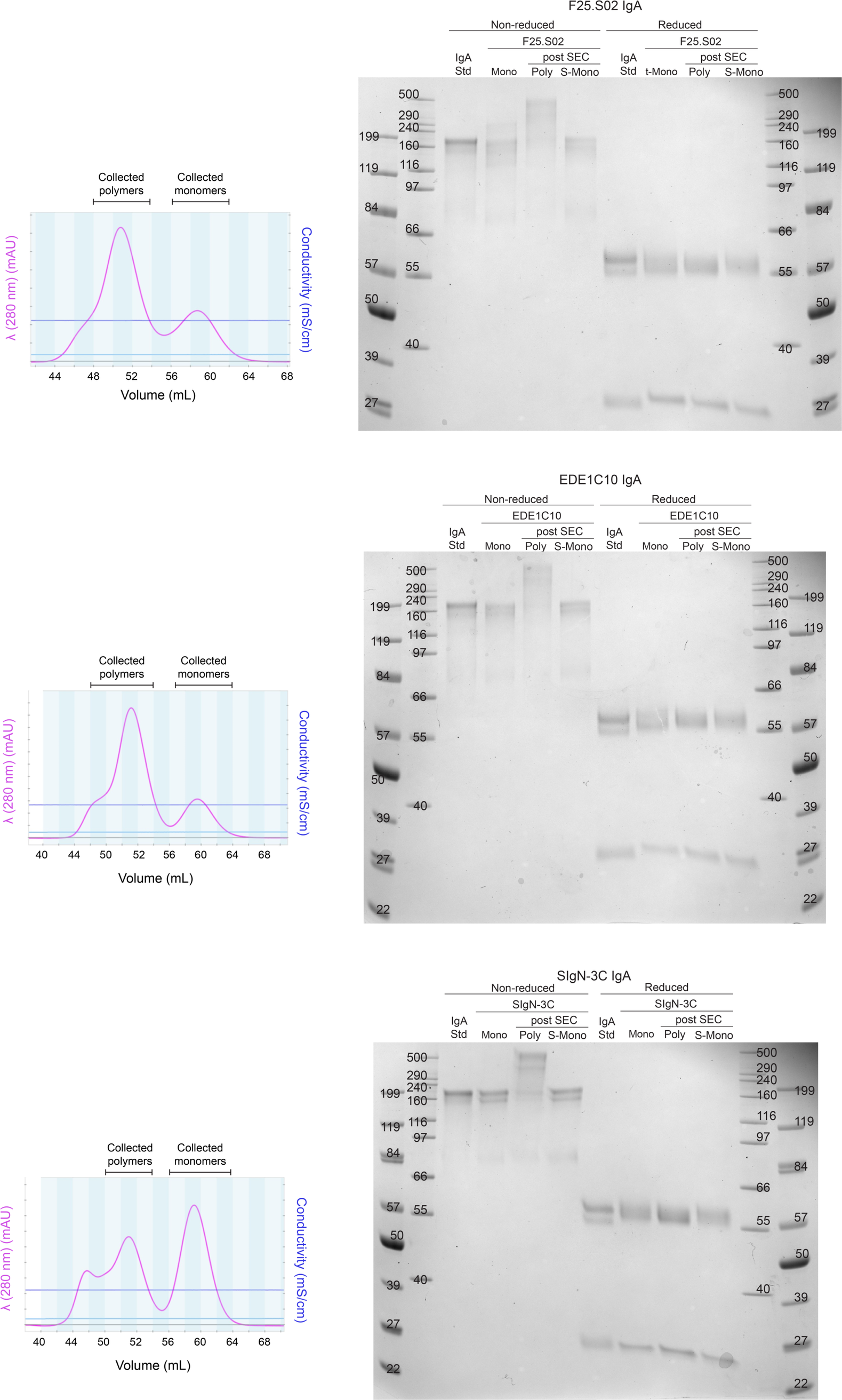

Supplement: S5 Fig — Graphs on the left display absorbance profiles (at 280 nm) of eluates from size-exclusion chromatography (SEC), which was used to separate monomeric and polymeric IgA1. Images on the right display SDS-PAGE gels to assess purity of preparations. Eluates from SEC were collected in 2 mL fractions and the fractions indicated were collected, pooled, and concentrated to obtain purified monomers and polymers. SDS-PAGE was run on non-reduced (left half) and reduced (right half) samples of each type of antibody. Each half of a gel has one well containing a commercially purchased IgA1 isotype control (IgA Std). Each half also has wells containing two types of IgA1 monomers. The first was produced as monomers, i.e in the absence of a J chain expression plasmid (Mono). The second were produced in a transfection that included a J chain expression plasmid and they were separated from polymers via SEC (S-Mono). Both types of monomers appeared similar by SDS-PAGE, but for simplicity all experiments were performed using the monomers produced in the absence of J chain. (TIF) [file ppat.1011722.s005.tif]

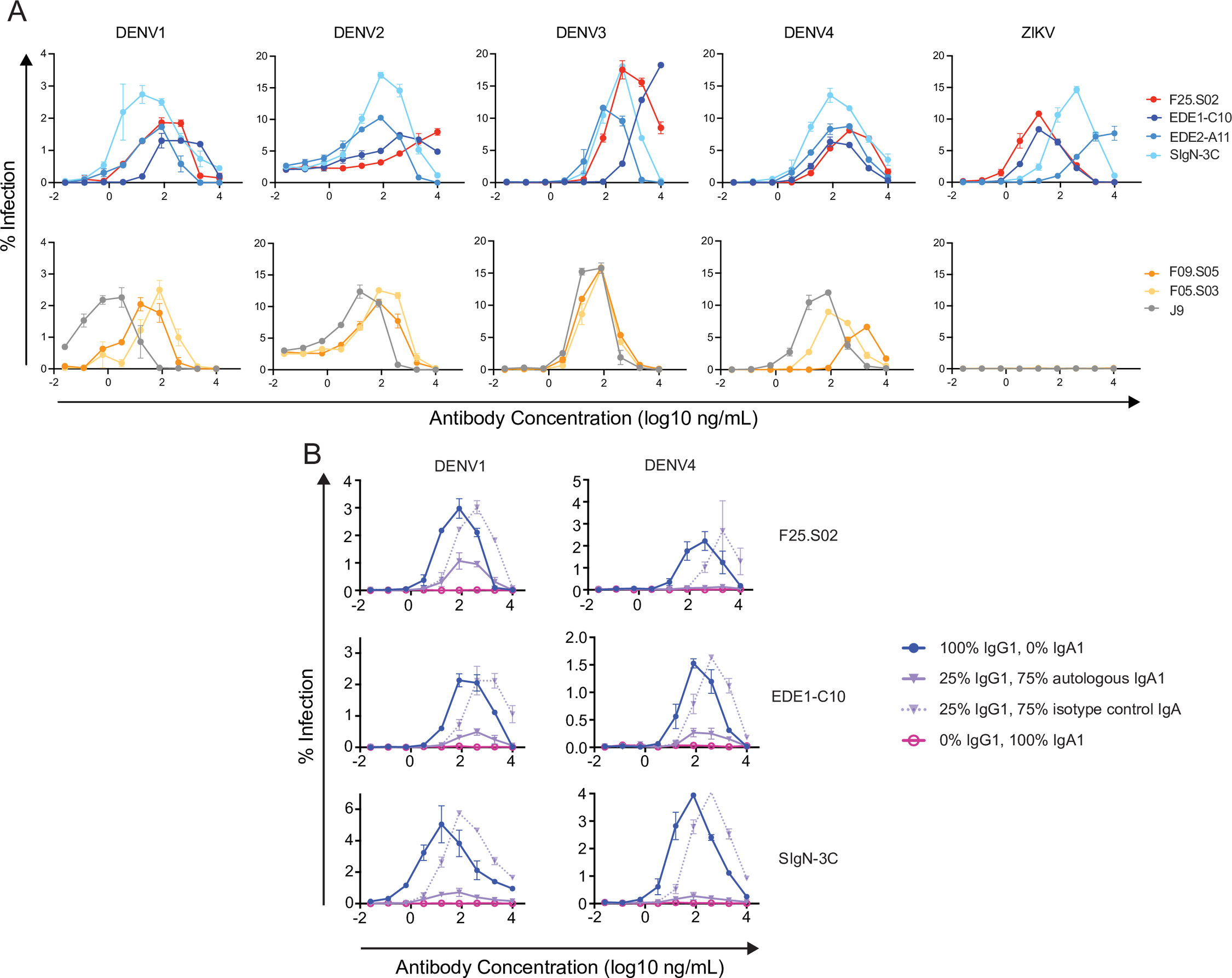

Supplement: S6 Fig — (A) Serial dilutions of IgG1 antibodies indicated in the key were complexed with DENV1 WP-74, DENV2 16681, DENV3 CH53489, DENV4 TVP376, and ZIKV H/PF/2013 reporter virus particles prior to infection of K562 cells, which express FcγRIIa but not FcαRI. Dose-response ADE profiles of antibodies that do or do not neutralize ZIKV in addition to DENV1-4 are shown in top and bottom panels, respectively. Data points and error bars indicate the mean and range of infection in duplicate wells, respectively. Graphs shown are representative of 4–5 independent experiments. (B) IgG1 and monomeric IgA1 forms of F25.S02 (top row), EDE1-C10 (middle row) or SIgN-3C (bottom row) were tested either individually or mixed at the indicated ratios by mass before serial dilution and pre-incubation with DENV1 WP-74 (left) or DENV4 TVP376 (right) reporter virus particles. Virus-antibody complexes were then used to infect K562 cells. Addition of isotype control IgA1 to IgG1 forms of each bnAb was included as controls. The experiment was performed in three biological replicates, each in duplicate wells. The data points and the error bars represent the means and the range of the duplicate wells, respectively, of one representative experiment. (TIF) [file ppat.1011722.s006.tif]

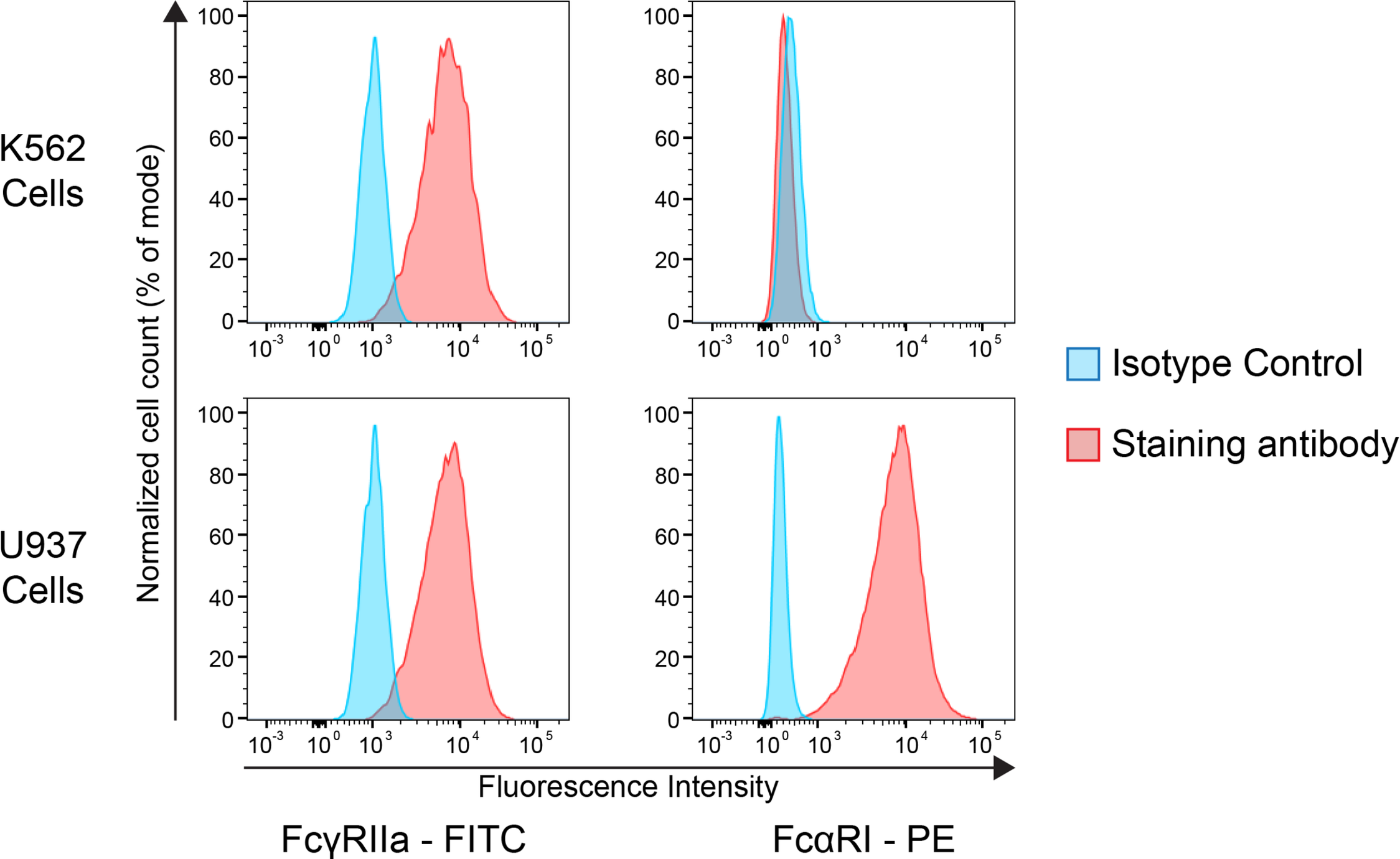

Supplement: S7 Fig — Histograms display the fluorescence intensity of K562 (top row) or U937 (bottom row) cells stained for the indicated Fc receptors. Histograms are normalized to the modal cell count. The isotype control was conjugated to the same fluorophore and used at the same concentration as anti-FcγRIIa or anti-FcαRΙ antibody on the same population of cells. (TIF) [file ppat.1011722.s007.tif]

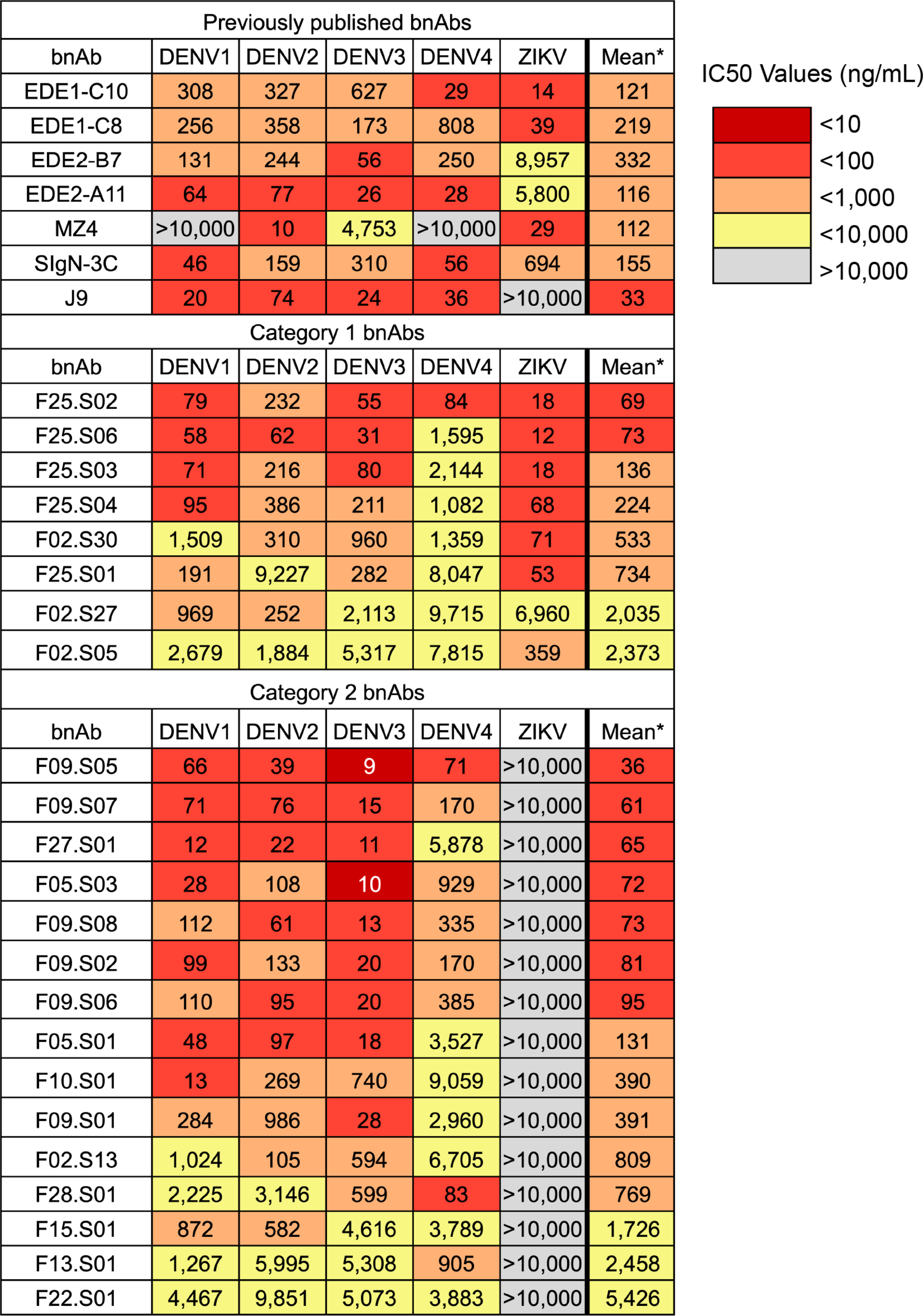

Supplement: S1 Table — For each virus, the value reported is the arithmetic mean IC50 from at least three independent experiments performed in duplicate. *Geometric mean IC50 for all neutralized viruses, i.e. values >10,000 ng/ml (the highest antibody concentration tested) were omitted. All antibodies were isolated from donor 014 except for F15.S01, which was isolated from donor 012. (TIF) [file ppat.1011722.s008.tif]

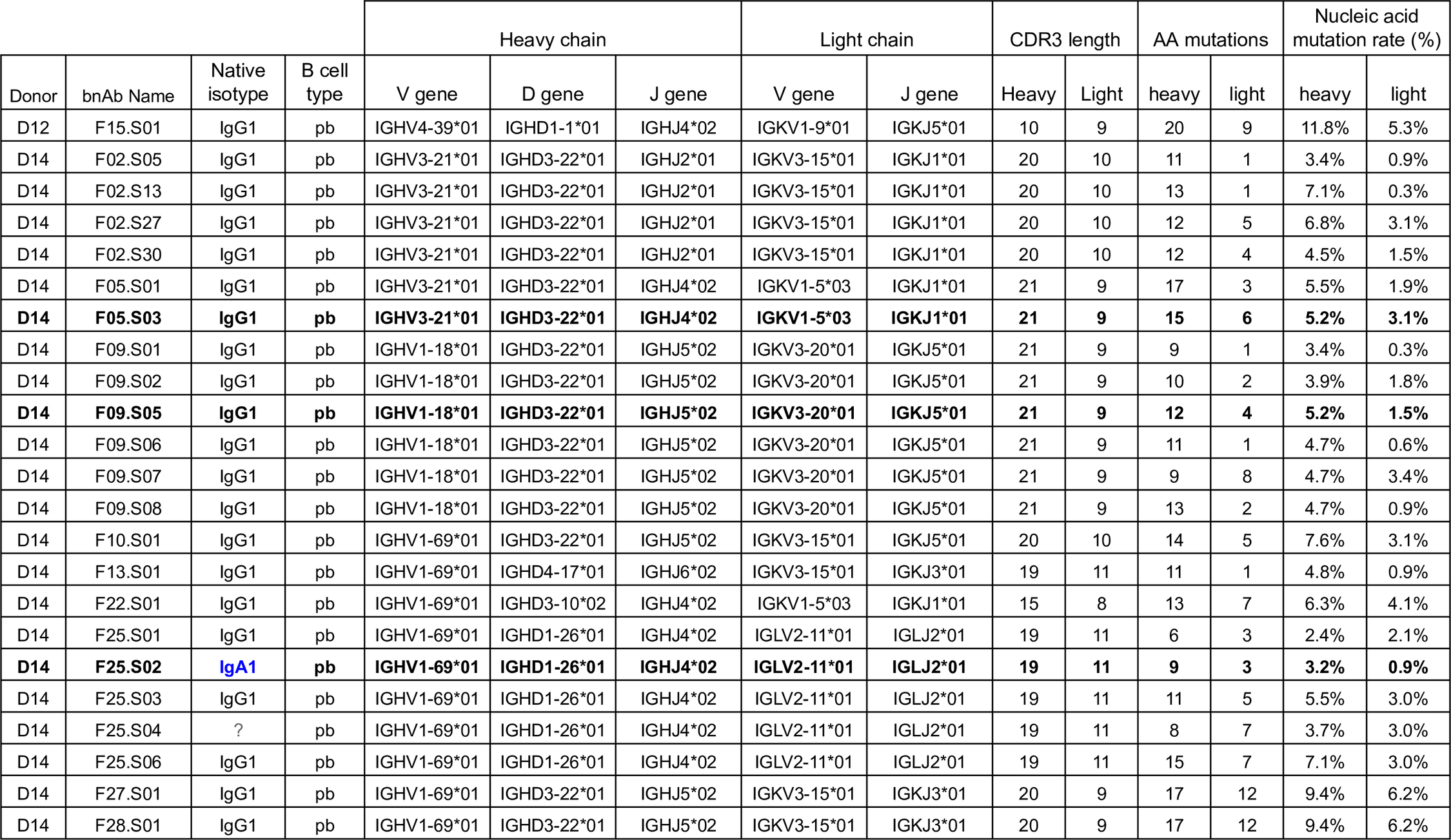

Supplement: S2 Table — Bold = chosen for detailed characterization; blue = non-IgG isotype; ? = insufficient sequence coverage of constant gene to determine the antibody’s isotype; pb = plasmablast. (TIF) [file ppat.1011722.s009.tif]

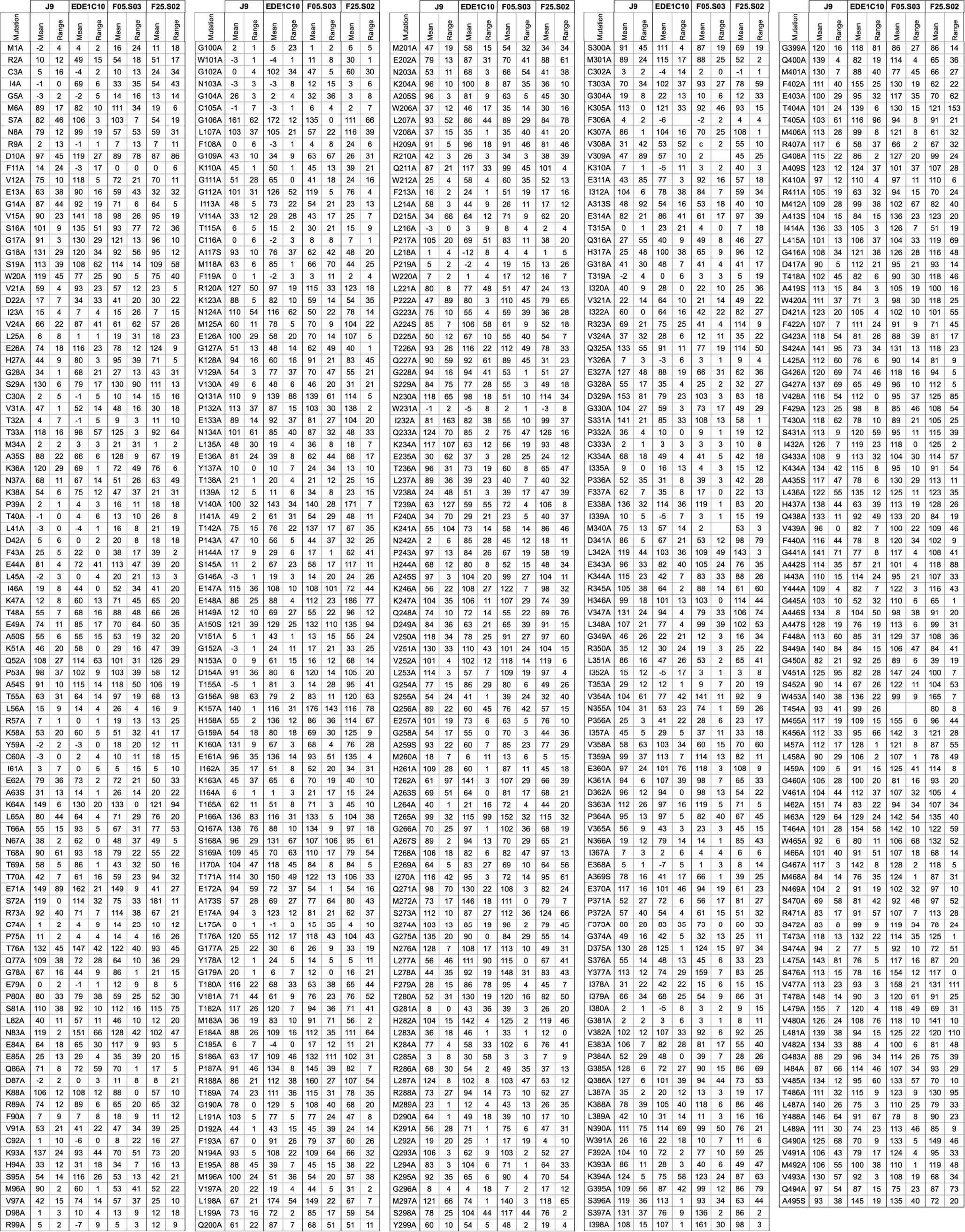

Supplement: S3 Table — Mean percentage and range of binding reactivity to alanine mutant relative to wild type DENV2 from at least two independent experiments. (TIF) [file ppat.1011722.s010.tif]

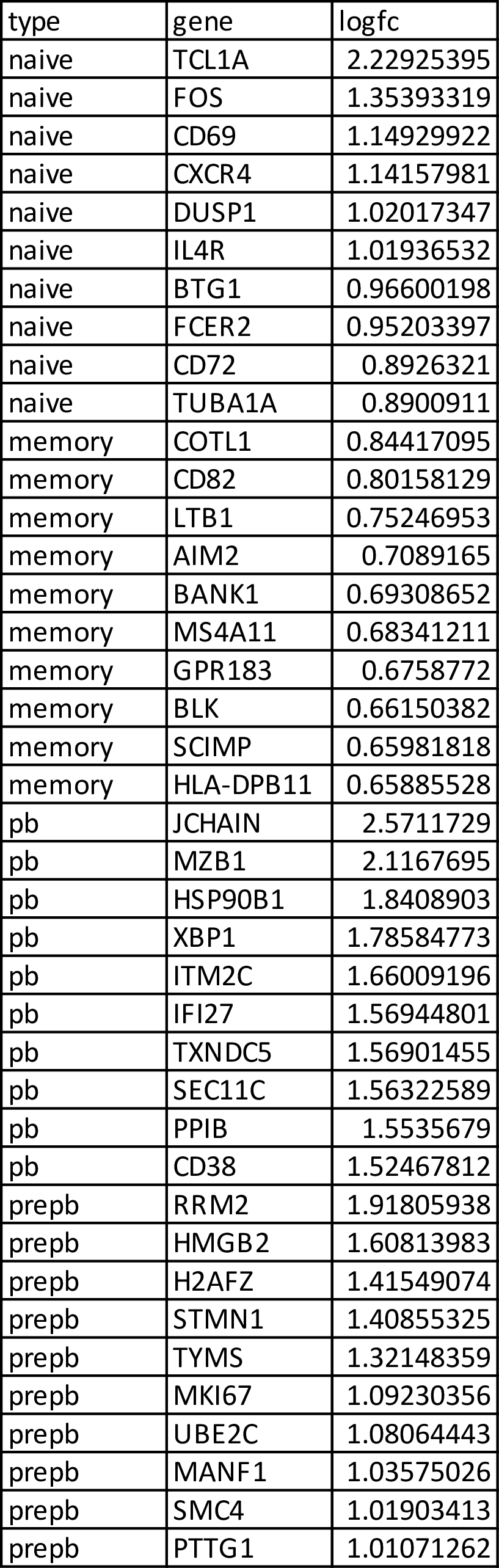

Supplement: S4 Table — (TIF) [file ppat.1011722.s011.tif]
