# Supplementary material for: Single B cell transcriptomics identifies multiple isotypes of broadly neutralizing antibodies against flaviviruses
Source: PLoS Pathog. 2023 Oct 9;19(10):e1011722. doi: 10.1371/journal.ppat.1011722 (PMC10586629; doi:10.1371/journal.ppat.1011722)
Supplement: Attachment — Submitted filename: response-PLoS-Path.docx [file ppat.1011722.s013.docx]

**Part I - Summary**

Reviewer #1: This manuscript by Lubow and colleagues report the identification of broadly neutralising antibodies against flaviviruses, in particular dengue viruses (DENVs) and that the IgA1 version of this antibody mediated neutralisation without enhancement, despite dilution. The authors conducted extensive studies to identify such antibodies and mapped their binding sites before exploring how IgG and IgA versions of antibodies with identical Fabs impact both DENV neutralisation and infection enhancement. This work adds to the very limited body of information on IgA antibodies against DENVs and suggest the potential of this type of antibody for therapeutic application.

We thank the reviewer for the positive comments.

Reviewer #2: This manuscript by Lubow et al. use scRNA-seq to identify broadly neutralizing antibodies (bnAbs) against dengue virus (DENV1-4) and Zika virus (ZIKV). The research utilizes an unbiased single-cell RNA sequencing (scRNAseq) approach of B cells, which allowed them to identify new bnAbs. This approach allowed the researchers to identify an IgA1 antibody (F25.S02) that exhibits potent cross-neutralizing activity against DENV1-4 and ZIKV. F25.S02 not only maintains its potency when expressed as IgG1 but also has unique binding and neutralization determinants compared to previously known classes of bnAbs. The study further shows that the IgA1 versions of bnAbs can inhibit IgG1-mediated antibody-dependent enhancement (ADE) in a dose-dependent manner. IgG antibodies have been the focus of flavivirus immunity studies, and these findings suggest an underappreciated role of flavivirus-specific IgA antibodies in infection and immunity. While the finding of the competitive role of IgA1 in preventing IgG induced ADE is interesting, there are some major gaps in how IgA is functioning, and the current data is a bit preliminary. Moreover, the B cell analyses in Figure 2 are very generalized and lack rigor in analysis. Lastly, the manuscript poorly references the data within the figures, making it difficult to connect data to the text.

We thank the reviewer for the comments and suggestions. Given the underappreciated role of IgA, we agree that there are ‘major gaps’ in our knowledge of its function in flavivirus immunity, which our study did not set out to investigate in detail. Instead, our main goal was to discover new flavivirus broadly neutralizing antibodies, which we accomplished using a method that captures the native isotypes of isolated antibodies. This method led us to unexpectedly identify the first IgA antibody that can broadly neutralize DENV1-4 and ZIKV. We are careful to acknowledge in our study that our *in vitro* findings of the role of IgA in ADE in particular are preliminary, and require validation in studies that are beyond the scope of this one. We address the reviewer’s critique on B cell analyses and manuscript flow in detail below.

**Note that due to a formatting issue, line numbers are different between the clean vs. tracked changes version of the revised manuscript. Line numbers described in our responses below refer to those in the clean version of the revised manuscript.**

**Part II - Major Issues**

Reviewer #1

1. The manuscript can greatly benefit from a revised narrative that focuses on the knowledge this study brings to flaviviral or even dengue immunity. In its present form, what knowledge Figures 2 and 3, along with the associated supplementary figures bring, is unclear. For instance, why was it that only donor 014 displayed the broadest neutralisation profile (lines 190-191)? Without knowing where the authors were going with the narrative, the very detailed description became a distraction. If there is indeed biological insights that can be drawn from this section of the manuscript, the description should be clearly framed to provide both context and interpretation. If not, the details could be best described in the methods or even supplementary section for improved flow of the narrative.

We agree with the reviewer that a more focused narrative is beneficial, especially with regards to Figure 2, in which we describe the distribution of B cell subsets and antibody isotypes within clonal families. We believe that a description of these data in the main results is appropriate as they inform our bioinformatics-based discovery of broadly neutralizing antibodies - the main goal of our study. To better focus our narrative on this main goal, we have substantially revised the content, order, and headings of the first few paragraphs of Results, in which we describe **Fig 1, Fig 2**, and **S1 Fig** (lines 110-217).

We emphasize that new knowledge on dengue immunity was not the goal of our study - the small sample size of 4 donors precludes the ability to make meaningful conclusions on dengue immunity. Rather, as mentioned above, our goal was to discover broadly neutralizing antibodies from these donors based on their ability to broadly and potently neutralize DENV1-4 and ZIKV, and on the availability of samples from which to isolate B cells. We have now revised the start of the last paragraph of the introduction to clarify this goal (line 99-101). In the discussion, we also acknowledge that we cannot make any conclusions on why donor 014 displayed the broadest neutralization profile as our study was not designed to investigate the basis for donor-dependent effects, though we provide some possible explanations (lines 517-531).

2. Line 115. Not all the viruses used in this neutralisation screen are prototypic. Please revise.

We have replaced ‘prototype’ with ‘commonly used’ in the relevant sentence (line 118).

3. Line 183. Were positive and negative controls included in this screen at a single dilution?

Yes, we produced and screened the previously published EDE1-C10 (positive control) and CR4354 (negative control) IgG1 antibodies in parallel with our test antibodies. All antibodies were screened against DENV1-4, ZIKV, and the more distantly related WNV. As expected, EDE1-C10-containing transfection supernatant neutralized DENV1-4 and ZIKV but not WNV, while CR4354-containing supernatant neutralized WNV but not DENV or ZIKV. Our original version of **S2 Figure** showed screening data for all downselected antibodies and control EDE1-C10 antibody against DENV1-4 and ZIKV, but not for CR4354 or WNV controls. We have now revised this figure to include the data for additional controls (**S2A Figure**) and included a description of these data in the corresponding results section (lines 194-199).

4. Figure 6B. The difference in IC50 between polymeric and monomeric IgA1 was greatest for F25.S02 against DENV2 and SIgN-3C against DENV3 and ZIKV. In contrast, although the rest also show lower IC50 with polymeric IgA1, they do not appear to be statistically significant. Are there any possible reasons for these findings?

We agree with the reviewer’s interpretation of the data. As noted in the discussion, defining the mechanism(s) behind this observation awaits further studies, but it suggests that depending on the virus, the bnAb epitopes are arranged in a manner that distinctly impacts multivalent engagement by polymeric IgA. We have revised the relevant section of the discussion to include this possible interpretation (lines 549-560).

Reviewer #2

1. It is not clear why the authors sorted out only CD19+ cells in order to find rare populations. The authors make a nice argument for why plasmablasts are a useful population to look at.

While our objective was to maximize the yield of plasmablasts, during our pilot experiments we found that when we sorted samples with fewer than 30,000 plasmablasts we had difficulty recovering enough cells to achieve the required density and volume for processing by the 10X chip. We therefore chose to enrich plasmablasts by sorting only if the sample met two criteria: 1) the fraction of B cells that were plasmablasts was <10%, and 2) the total number of plasmablasts in the sample was > 30,000. Only one donor’s sample (002) met both criteria, so it was the only sample we sorted. We have revised the methods section to include these details (lines 643-652).

2. Line 155, the authors conclude that Donors 002 and 014 have more plasmablasts than donors 001 and 012. While this appears to be true, there are lot of plasmablast clonal expansions in 001 and 012, but are presented on a different scale. It should also be noted that the authors crudely sorted on CD19+ cells and as a result, it’s not surprising there are a lot of naïve B cells.

Our statements about the frequency of plasmablasts are made relative to other B cell subsets within the sample, not about the absolute number of plasmablasts in any given patient’s blood. Therefore, we chose axes that facilitated comparisons of family size, B cell type, and antibody isotype within each sample, not across samples. As described in our response to Reviewer 1, the objective of Fig 2 is to inform readers of the analyses of the samples leading to our downselection of candidate bnAbs, not to characterize B cell responses to DENV generally. As noted above, we have now significantly revised this section to focus the narrative on our objective (lines 110-217)

3. Figure 2 very crudely parses out the distinct B cell subsets, with limited details about the bioinformatics on how this done, fidelity of subsetting, etc. Further interrogation and analyses presented would help with this conclusion.

We have now added additional details about how B cell subsets were assigned in the methods section (lines 676-680) and included the list of reference genes used for this assignment in the **new S4 Table**.

4. Paper generally poorly references data. For example, in the paragraph starting at line 181, a reference to data is only made once. Paragraph starting at Line 212 doesn’t even link to data in the paper.

We thank the reviewer for this critique. The paragraphs cited refer to data presented in **S2 Fig** and **S1 Table** (revised submission lines 189-243). We have now added panel labels to S2 Fig to connect the text more clearly to the relevant data in the figure. We have also simplified our heatmap presentation in this figure. Throughout this section and the rest of the manuscript, we have also ensured references to specific figure panels appear immediately following the corresponding description in the text.

5. Fig. S2. The use of crude supernatants for neutralization experiments without standardizing for antibody is not recommended. What if a clone expresses better and as a result, this one is selected for downstream analysis when there is a better antibody in the dataset?

We appreciate the reviewer’s point that a negative result in our initial screen may not accurately predict neutralizing activity. We have now added this caveat to the discussion (lines 602-606). As with any approach, we agree our method of using crude supernatant in our initial round of screening has limitations and likely missed some neutralizing antibodies that express poorly. However, we considered this an acceptable tradeoff given the extra time, labor, and cost associated with purifying and quantifying each antibody screened. The initial round of recombinant antibody production and screens were performed by only two researchers (the first and second authors). We therefore concluded that a faster, broader search using crude supernatant was more beneficial than a slower, narrower search using purified antibodies, which we did perform with promising antibodies in downstream steps. Thus, while there may be ‘better’ antibodies yet to be discovered, we argue that despite its limitations, this approach achieved our goal of discovering novel flavivirus bnAbs, including the first non-IgG isotype that can potently and broadly neutralize DENV1-4 and ZIKV.

6. Line 200, hard to make this conclusion without knowing the number of mAbs made.

Line 200 in the original manuscript (Line 216 in revised manuscript) says, “These results demonstrate that our bioinformatics-based approach successfully identified clonal families encoding multiple bnAbs.” This conclusion is supported by the data described in this section and presented in **S2B-G Figs**, which also indicate the number of antibodies screened from each donor. Importantly, we do not (and cannot) make any conclusions on whether our method is more or less efficient than others at finding bnAbs.

7. Figure 7 and associated text, is IgA+virus able to engage FcaRI? FcaRI is a low affinity receptor, as a result requires cross-linking to induce engagement. Does dimeric (or polymeric) IgA lead to ADE?

We have added the requested new data to **Fig 7A** and described it in the corresponding results section in lines 476-478: *“Although we obtained the canonical dose-response ADE curve using IgG1 versions of bnAbs, neither monomeric nor polymeric IgA1 antibodies enhanced DENV infection in U937 monocytes (Fig 7A).”*

8. Does the competition between IgG and IgA occur in the K562 cell line?

As observed in U937 cells, ADE mediated by IgG1 in K562 cells was inhibited by autologous monomeric IgA1 versions of bnAbs F25.S02, EDE1-C10, and SIgN-3C, but not by isotype control IgA. We have added this new data to **S6B Fig** and described it in the corresponding results section (lines 478-487).

**Part III - Minor Issues**

Reviewer #1

1. Line 269. There is no fifth DENV serotype. Despite the claims of one of the authors, the papers that examined this DENV strain concluded that it was a divergent DENV4, which is not surprising since this is a sylvatic virus. Please revise.

We have removed the reference to a fifth serotype and revised our description of DKE-121 as “a recently described, highly divergent DENV4 strain” (line 279).

2. Lines 310-311 and Fig S1. The viral strains described in the methods section are inconsistent with those reported in Fig S1.

We found it difficult to pinpoint the inconsistency the reviewer was referring to here. Lines 310-311 of the initial manuscript are in the legend for Figure 4. They do not relate to S1 Fig. We found two possible sources of confusion, which we clarify as follows:

1) It is possible that the reviewer meant to cite lines 810-811 of the initial submission, which is in the methods section describing “Neutralization and antibody-dependent enhancement assays using reporter virus particles” (revised submission lines 828-849). Here, we listed all reporter virus strains relevant to not only **S1 Fig** but also **Fig 3**. We have now revised this section to specify which reporter virus strains were used for which figures (lines 831-835). We also noticed the strain of WNV used (NY99) in S1 Fig (and revised S2 Fig) was accidentally omitted in this section of the initial submission; we have now added this information in the revised submission (line 831).
2) In Fig 3, we show neutralization data for not only single-round infectious reporter viruses described above, but also for fully infectious virus strains. We describe the fully infectious virus strains in a separate methods section entitled “Production and neutralization of fully infectious viruses” (initial submission lines 826-829, revised submission lines 850-869). It is possible that the reviewer mistakenly thought the fully infectious virus strains described in this section correspond to those for reporter viruses in S1 Fig.

Reviewer #2

1. Lines 120-121 – the authors mention that “the high-prevalence of cross-serotype neutralizing antibodies likely reflects repeated DENV exposures, as confirmed by IgG avidity testing” and then reference a few papers. The way this is written makes it sound like they are doing this in the study, but its actually data from other studies. I would recommend rewording this to make sure it is clear that this already published data.

We have revised the sentence as follows to clarify that IgG avidity testing was performed in prior studies (lines 124-125): *“This high prevalence of cross-serotype neutralizing activity likely reflects repeated DENV exposures, as confirmed by IgG avidity testing of these samples in prior studies [42,43].”*

2. Line 459, lack of ADE by those mAbs are due to the antibodies being unable to neutralize or to bind altogether? This is a bit confusing given that in the introduction that it was proposed non-neutralizing antibodies are responsible for ADE.

As we do not refer to any antibodies in line 459, we assume the reviewer is referring to lines 460-461 in the initial submission where we describe lack of enhancement of ZIKV infection in FcgR-expressing K562 cells by antibodies J9, F09.S05, and F05.S03 (**S6A Fig**). As K562 cells are permissive to infection only by IgG-bound virions, the fact that these IgG antibodies failed to enhance ZIKV infection suggests an inability to bind to the virus, at least across the range of antibody concentrations tested in the ADE assay. This lack of binding could explain their inability to neutralize ZIKV (**Fig 3**). We have now clarified this section (lines 463-465 in revised submission).

3. Patient information just references two other papers. This should be included within the manuscript.

Patient information (age, type of acute exposure (DENV or ZIKV), infecting DENV serotype (if known), and sample collection time point) is already included in **S1 Fig**. We have now highlighted the availability of this information in the main text (lines 119-120).

Other revisions

- In the original manuscript, Fig 7A compared the ability of IgG1 and IgA1 versions of bnAbs to mediate ADE in K562 cells. In the revised manuscript, we have replaced this figure with the additional experiment requested by Reviewer 2, i.e. ADE assays performed with IgG1, monomeric IgA1, and polymeric IgA1 versions of bnAbs on U937 cells.
- Data for all ADE experiments performed in K562 cells are now displayed in revised S6 Figure. Panel B of this figure shows data for the additional IgG/IgA competition experiment requested by Reviewer 2.
- Due to an error in the spreadsheet used to calculate the mean IC50 values displayed in Fig 3C, the initial submission listed the IC50 values of F25.S02 and EDE1-C10 against DENV4 strain H241 as 70 ng/mL and 20 ng/mL, respectively. We fixed the error in the formula and the revised version of Fig 3C has the correct values (113 ng/mL and 37 ng/mL). This error does not impact our conclusions based on the data presented, namely that category 1 bnAbs (such as F25.S02 and EDE1-C10) but not category 2 bnAbs retained neutralization potency against this genotype I DENV4 strain (lines 275-276).
