# Supplementary material for: Single B cell transcriptomics identifies multiple isotypes of broadly neutralizing antibodies against flaviviruses
Source: PLoS Pathog. 2023 Oct 9;19(10):e1011722. doi: 10.1371/journal.ppat.1011722 (PMC10586629; doi:10.1371/journal.ppat.1011722)
Supplement: Attachment — Submitted filename: response2-PLoS-Path.docx [file ppat.1011722.s014.docx]

Reviewer #1: The authors have addressed all the concerns raised in their original manuscript. This paper now tells a logical and interesting story on an alternative method to discovery broadly neutralising antibody against DENV and ZIKV, as well as the under-appreciated role of IgA antibodies in the adaptive immune response against such flaviviral infections.

We thank the reviewer for the positive comments and helpful suggestions to improve the manuscript.

Reviewer #2: The authors have addressed all of my concerns except for their response to major concern #5.
I do not find the authors argument compelling in the lack of standardization. I appreciate that purifying individual antibodies is time consuming, but it is also not challenging. That said, if the authors insist on using supernatants, they need to quantify the amount of antibody within that supernatant, which is a single and straightforward ELISA, and standardize their results based on this. I understand the point of trying to find neutralizing antibodies, but this approach and data presentation is not sound for publication and sets a bad precedent.

We have quantified the amount of IgG1 antibodies in each supernatant by ELISA and included this data in a new column in S2 Fig. In this figure, we display IgG1 levels as a blue heatmap side-by-side with the red heatmap corresponding to virus neutralization. With the data presented this way, we hope the reviewer can now appreciate that in most cases, lack of neutralizing activity was not simply explained by poor IgG1 expression. We have revised the end of our discussion (where we acknowledge study limitations) to include this interpretation (lines 603-609). We have also added a description of the ELISA to quantify IgG1 antibodies to the methods section (lines 761-7*73*).
